# A comparison of two PCR protocols for the differentiation of *Plasmodium ovale* species and implications for clinical management in travellers returning to Germany: a 10-year cross-sectional study

**DOI:** 10.1186/s12936-019-2901-0

**Published:** 2019-08-09

**Authors:** Hagen Frickmann, Christine Wegner, Stefanie Ruben, Ulrike Loderstädt, Egbert Tannich

**Affiliations:** 1 Department of Microbiology and Hospital Hygiene, External Site at the Bernhard Nocht Institute, Tropical Microbiology and Entomology, Bundeswehr Hospital Hamburg, Bernhard Nocht Str. 74, 20359 Hamburg, Germany; 2 Institute for Medical Microbiology, Virology and Hygiene, University Medicine Rostock, Rostock, Germany; 3 Bernhard Nocht Institute for Tropical Medicine, National Reference Center for Tropical Pathogens, Hamburg, Germany

**Keywords:** Malaria, *Plasmodium ovale curtisi*, *Plasmodium ovale wallikeri*, Parasite latency, Epidemiology, Test comparison

## Abstract

**Background:**

To assess the occurrence of *Plasmodium ovale wallikeri* and *Plasmodium ovale curtisi* species in travellers returning to Germany, two real-time PCR protocols for the detection and differentiation of the two *P. ovale* species were compared. Results of parasite differentiation were correlated with patient data.

**Methods:**

Residual nucleic acid extractions from EDTA blood samples of patients with *P. ovale* spp. malaria, collected between 2010 and 2019 at the National Reference Centre for Tropical Pathogens in Germany, were subjected to further parasite discrimination in a retrospective assessment. All samples had been analysed by microscopy and by *P. ovale* spp.-specific real-time PCR without discrimination on species level. Two different real-time PCR protocols for species discrimination of *P. o. curtisi* and *P. o. wallikeri* were carried out. Results were correlated with patient data on gender, age, travel destination, thrombocyte count, and duration of parasite latency.

**Results:**

Samples from 77 *P. ovale* spp. malaria patients were assessed, with a male:female ratio of about 2:1 and a median age of 30 years. Parasitaemia was low, ranging from few visible parasites up to 1% infected erythrocytes. Discriminative real-time PCRs revealed 41 cases of *P. o. curtisi* and 36 cases of *P. o. wallikeri* infections. Concordance of results by the two PCR approaches was 100%. Assessment of travel destinations confirmed co-existence of *P. o. curtisi* and *P. o. wallikeri* over a wide range of countries in sub-Saharan Africa. Latency periods for the two *P. ovale* species were similar, with median values of 56.0 days for *P. o. curtisi* and 58.0 days for *P. o. wallikeri*; likewise, there was no statistically significant difference in thrombocyte count with median values of 138.5/µL for patients with *P. o. curtisi* and 152.0/µL for *P. o. wallikeri*-infected patients.

**Conclusions:**

Two different real-time PCR protocols were found to be suitable for the discrimination of *P. o. curtisi* and *P. o. wallikeri* with only minor differences in sensitivity. Due to the overall low parasitaemia and the lack of differences in severity-related aspects like parasite latency periods or thrombocyte counts, this study supports the use of *P. ovale* spp. PCR without discrimination on species level to confirm the diagnosis and to inform clinical management of malaria in these patients.

**Electronic supplementary material:**

The online version of this article (10.1186/s12936-019-2901-0) contains supplementary material, which is available to authorized users.

## Background

Human *Plasmodium ovale* spp. malaria is caused by two different sympatric *Plasmodium* species, namely *P. ovale curtisi*, which was considered the “classic” human pathogen, and *P. ovale wallikeri*, which has been described as a genetically distinguishable variant. Parasitological features such as parasitaemia, 18S rRNA gene copy number, or pan-aldolase activity are similar, with an overrepresentation of infected erythrocytes lacking Schüffner’s stippling among *P. ovale wallikeri* isolates [1]. Although the organisms are morphologically virtually indistinguishable and are associated with sympatric ranges through tropical Africa, Asia, and Oceania, considerable genetic differences have been observed between *P. ovale curtisi* and *P. ovale wallikeri*. They do not recombine and are associated with substantial genetic divergence between sexual stage proteins, such as 6-cysteine and proteins containing Limulus coagulation factor C domains, probably resulting in sexual incompatibility [2]. Phylogenetic separation and diversity of *P. o. curtisi* and *P. o. wallikeri* surface antigens support species status [3, 4].

The occurrence of *P. o. curtisi* and *P. o. wallikeri* in returning travellers has been reported from Canada [1], China [5–12], Southern Europe [13, 14], and the UK [15]. A wide range of sub-Saharan Africa has been identified as the major region of *P. ovale* spp. acquisition for Chinese travellers [8, 9, 11, 12], while a majority (88%) of returnees to Canada came from Western Africa [1]. Both *P. ovale* spp. have been infrequently reported from Bangladesh [16]; Bioko Island [17], the Comoros Islands [18], Congo-Brazzaville [17], Ethiopia [19, 20], Ghana [21], India [22], the Ivory Coast [16], Kenya [23], Senegal [24, 25], and Uganda [17]. *Plasmodium o. wallikeri* is also prevalent in Malaysia [26], while *P. o. curtisi* infections have recently been reported from Sri Lanka [27]. Interestingly, *P. o. wallikeri* has been detected both in human patients and in Western Lowland gorillas in the Central African Republic [28].

Potential differences in pathogenicity of *P. o. curtisi* and *P. o. wallikeri* are of ongoing interest. An early Spanish study assessing patients from 2005 to 2011 indicated significantly more severe thrombocytopenia in patients with *P. o. wallikeri* malaria as well as non-significant trends for shorter time periods between infection and onset of clinical symptoms (latency period), lower albumin levels, higher temperature, and more haemolysis markers compared to patients with *P. o. curtisi* infections [13]. A British study showed geometric mean latency periods of 40.6 days for *P. o. wallikeri* and of 85.7 days for *P. o. curtisi*, with both parasites frequently occurring in patients despite their using chemoprophylaxis during travel compared with *Plasmodium vivax* or *Plasmodium falciparum* malaria [15]. A multi-centre study conducted in France, Italy, and Spain recently also suggested *P. o. wallikeri* to be more pathogenic, as defined by associations with more severe thrombocytopenia, coagulopathy as expressed by higher international normalized ratios (INR), and shorter latency in Caucasians.

Further, *P. o. wallikeri* infections were more frequent in males and particularly in Caucasians. However, severe cases were observed for *P. o. curtisi* as well, and infections with both *P. o.* spp. suggested an association with diabetes mellitus and—again—decreased effectiveness of anti-malarial drugs as chemoprophylaxis compared with *P. falciparum* [14]. The latter phenomenon might be associated with the geometric mean latency periods and the formation of hypnozoites. Recently, a well-characterized case of a hypnozoite-associated relapse of *P. o. wallikeri* malaria with a strain of identical genotype has been reported [29]. However, few well-documented cases of *P. ovale* spp. malaria relapses have been published so far [29–31] with a strong dominance of *P. o. curtisi* [31].

Various PCR approaches for the detection and discrimination of *P. o. curtisi* and *P. o. wallikeri* have been described [18, 32–36], including TaqMan qPCR [18, 32–34], semi-nested PCR [37], and nested PCR [38] as well as quantitative and high-resolution melting approaches with detection limits as low as 1 parasite/µL [39]. Multilocus genotyping has also been applied for *P. o. curtisi* and *P. o. wallikeri* discrimination [23, 38].

In this study, samples from 77 patients with *P. ovale* spp. malaria returning to Germany between 2010 and 2019 were further differentiated in a retrospective assessment based on two different real-time PCR approaches. Results were compared with patient characteristics to contribute to the scant information available on the possibly differences in aetiological relevance of *P. o. curtisi* and *P. o. wallikeri*.

## Methods

### Aim, design, and setting

The epidemiology of *P. o. curtisi* and *P. o. wallikeri* in travellers returning to Germany and the available patient-related information were assessed in a retrospective cross-sectional study over 10 years using clinical information and residual sample materials collected at the Bernhard Nocht Institute for Tropical Medicine Hamburg, the German National Reference for Tropical Pathogens, between 2010 and 2019.

### Samples

Residual nucleic acid extractions from EDTA blood samples of all 77 patients with confirmed *P. ovale* spp. infections within the study interval were used, which had been collected and stored at − 80 °C at the Bernhard Nocht Institute for Tropical Medicine, Hamburg, Germany. The samples were collected between 2010 and 2019. Extractions had been performed using the QIAamp DNA Blood Mini Kit (Qiagen, Hilden, Germany). All samples had been assessed by species-specific real-time PCR before storage (RealStar Malaria S&T PCR Kit 1.0, altona Diagnostics, Hamburg, Germany), and a subset also by microscopy (Additional file 1).

### PCR-based discrimination of *P. ovale curtisi* and *P. ovale wallikeri*

All DNA samples were analysed using two different published real-time PCR protocols suitable for the discrimination between *P. o. curtisi* and *P. o. wallikeri*. PCR protocol 1 was applied as a duplex real-time PCR using the primers for *P. o. curtisi* and *P. o. wallikeri* as described by Bauffe et al. [18] (Additional file 2). The reaction was run on a Corbett RotorGene 6000 or a Corbett RotorGene Q cycler using 20 µL volumes with a HotStarTaq Mastermix (Qiagen). The final MgCl_2_ concentration was adjusted to 3 mmol/L; the applied primer concentrations were 1.6 pmol/µL and the probe concentration 0.1 pmol/µL each. A 5 µL volume of extracted DNA was used. Reaction conditions comprised 10 min at 95 °C followed by 45 cycles of 10 s denaturation at 95 °C as well as annealing and amplification for 30 s at 60 °C with subsequent cooling to 40 °C for 30 s.

PCR protocol 2 consisted of two separate real-time PCRs, one specific for *P. o. curtisi* and the other specific for *P. o. wallikeri* as described by Calderaro et al. [32, 33] (Additional file 2). Again, 20 µL volumes were assessed on a Corbett RotorGene 6000 or a Corbett RotorGene Q cycler using the HotStarTaq Mastermix (Qiagen). The optimal final MgCl_2_ concentration was 3 mmol/L; the primer concentrations were adjusted to 1.2 pmol/µL and the probe concentration to 0.25 pmol/µL each. The amount of extracted DNA used was 2 µL. Reaction conditions comprised 10 min at 95 °C followed by 45 cycles of 15 s denaturation at 95 °C as well as annealing and amplification for 60 s at 60 °C with subsequent cooling to 40 °C for 30 s.

The oligonucleotide sequences of the primers, probes, and positive control plasmids are shown in Additional file 2. The positive control plasmids based on pEX-A128 vector backbones (eurofins Genomics, Ebersberg, Germany) were used to prepare rows of 10-fold dilutions in order to calculate detection limits using the program SciencePrimer (http://scienceprimer.com/copy-number-calculator-for-realtime-pcr, last accessed March 8, 2019). For PCR protocol 1, calculated detection limits were < 1 copy/µL for *P. o. wallikeri* and 52 copies/µL for *P. o. curtisi*; for PCR protocol 2, calculated detection limits were 8 copies/µL for *P. o. wallikeri* and 9 copies/µL for *P. o. curtisi*.

Inhibition control PCRs were not included in these typing PCRs, because inhibition had already been excluded during the initial diagnostic PCR that led to the diagnosis of *P. o.* spp. infection.

### Patient characteristics

All patients were anonymized and the respective clinical data were computer-coded. As far as available, the following data were assessed: gender, age, region of presumed infection, number of days between leaving the endemic country and onset of malaria symptoms, and thrombocyte counts.

### Statistics

Due to the low number of *P. ovale* spp. positive samples, only descriptive analysis was performed. The Mann–Whitney test was applied to independent parameters as age, latency time, and thrombocyte count using the GraphPad Instat version 3.06 software (GraphPad Software, Inc., San Diego, CA, USA). Matched parameters as Ct-values while comparing both PCR assays were assessed using Wilcoxon’s signed ranks test with the help of the open-source software R Commander version 2.5–3, which was also used to calculate descriptive parameters like interquartile ranges (IQR).

### Ethics

Ethical clearance was provided by the Ethics Committee of the Medical Association of Hamburg, Germany (registration number WF-012/19), allowing the anonymized assessments to be made without informed consent.

## Results

### Samples

Residual DNA from 82 diagnostic EDTA blood samples containing parasites of *P. ovale* spp. according to the results of microscopy and PCR was stored at the German National Reference Center for Tropical Pathogens Bernhard Nocht Institute for Tropical Medicine Hamburg (BNITM). The samples had been sent to BNITM from clinicians and laboratories all over Germany for diagnostic verification. Of the 82 samples, 5 represented follow-up samples. Accordingly, first diagnostic samples from 77 independent patients were included in this study. Parasitaemia was generally low and ranged from a few visible parasites up to 1% infected erythrocytes. Initial microscopic identification of *P. ovale* spp. had been successful in 12 samples, while initial misidentification as *P. vivax* had occurred in 3 cases. In the remaining 67 samples, either parasitaemia was insufficient for a qualified microscopic parasite differentiation (n = 40) or microscopy had not been performed at the Bernhard Nocht Institute because PCR was requested by the sender of the samples only and the initial microscopic results from the senders’ laboratories were unknown (n = 17) (Table 1).

**Table 1:** Summary of the diagnostic results (details in Additional file 1)

Microscopic species identifications	12× *P. ovale* spp., 3× misidentifications as *P. vivax*, 40× *Plasmodium* spp. without further discrimination, 17× not performed
Range of parasitaemia in microscopically positive samples	Few visible parasites up to 1% infected erythrocytes
Ct values of the *P. ovale curtisi* PCR according to Bauffe et al. (median; IQR)	24; 3
Ct values of the *P. ovale curtisi* PCR according to Calderaro et al. (median; IQR)	26; 3
Ct values of the *P. ovale wallikeri* PCR according to Bauffe et al. (median; IQR)	25; 4
Ct values of the *P. ovale wallikeri* PCR according to Calderaro et al. (median; IQR)	28; 4.3

IQR = interquartile range

### Results of the *P. ovale wallikeri* and *P. ovale curtisi* PCRs

All 77 *P. ovale* spp. samples were subjected to two different real-time PCR protocols as previously reported [18, 32, 33]. The protocols showed 100% concordance and identified 41 patients with *P. o. curtisi* and 36 patients with *P. o. wallikeri*. Cycle threshold (Ct) values for *P. o. curtisi* were 25.4 ± 3.8 (mean ± standard deviation (SD)) with a median of 24 (IQR = 3) using the PCR of Bauffe et al. and 27.5 ± 3.9 (mean ± SD) with a median of 26 (IQR = 3) for the PCR of Calderaro et al. (P < 0.001). For *P. o. wallikeri*, the Ct values were 24.9 ± 5.3 (mean ± SD) with a median of 25 (IQR = 4) using the PCR of Bauffe et al. and 28.6 ± 4.2 (mean ± SD) with a median of 28 (IQR = 4.3) using the PCR of Calderaro et al. (P < 0.001). Differences in Ct value between the two PCR protocols were 1.9 ± 1.0 (mean ± SD) with a median of 2 (ranging from 0 to 3, IQR = 2) for *P. o. curtisi* and 3.6 ± 2.3 (mean ± SD) with a median of 3 (ranging from − 4 to 11, IQR = 2) for *P. o. wallikeri* (P < 0.001) (Table 1). Of note, in the five excluded follow-up samples, the Calderaro et al. approach failed to identify one case of *P. o. wallikeri* in a post-treatment control that was still positive in the Bauffe et al. approach, but with a relatively high Ct value of 37.

### Patient characteristics

Of the 77 patients included in the study, gender information was available for 76 participants. Altogether, 53 were male and 23 female. Male-to-female ratios differed considerably between the two groups of *P. o.* spp. infections comprising 3.4 and 1.6 for *P. o. curtisi* and *P. o. wallikeri*, respectively. Age information was available for 74 patients. Mean and median age were 31.6 ± 14.8 and 29.5 years (IQR = 23.5 years), respectively, and did not differ significantly between the two patient groups (P = 0.72). Mean age of *P. o. curtisi*-infected patients was 32.5 years with a standard deviation of 15.3 years and was 30.0 ± 10.8 years for *P. o. wallikeri* patients; the median age in both subgroups was 30 years (IQR = 26.0 years for *P. o. curtisi* and 20.5 years for *P. o. wallikeri*) (Table 2).

**Table 2:** Characteristics of patients with *P. ovale curtisi* or *P. ovale wallikeri* (details in Additional file 1)

	Patients with *P. ovale curtisi*	Patients with *P. ovale wallikeri*
Male-to-female ratio	3.4	1.6
Age in years (median; IQR)	30; 26.0	30; 20.5
Latency period in days (median; IQR)	56.0; 153.5	58; 98.0
Thrombocyte count per µL (median, IQR)	138.5; 105.8	152.0; 155.0

*IQR* interquartile range

Information on travel destinations was documented for 64 patients (32 with *P. o. curtisi*, 32 with *P. o. wallikeri*). Results are shown in Table 3 as well as in Additional files 1 and 3. No proven case of malaria relapse was documented.

**Table 3:** Patients’ travel destinations. Data were available for 32 patients with *P. ovale curtisi* and 32 patients with *P. ovale wallikeri*, (n) describes the numbers of patients with respective travel destinations

Travel destination	Patient with *P. ovale curtisi* (n)	Patient with *P. ovale wallikeri* (n)
Benin	–	1
Burkina Faso	–	1
Cameroon	3	7
Equatorial Guinea	1	–
Gabon	–	1
Ghana	5	6
Guinea	2	–
Ivory coast	1	3
Kenya	1	1
Liberia	2	–
Malawi	1	1
Nigeria	5	2
Papua New Guinea	–	1
Zambia	1	–
Sierra Leone	1	1
Somalia	1	–
Sudan	–	1
Tanzania	1	2
Togo	–	1
Uganda	3	2
Various destinations	4^a^	1^b^

^a^Various destinations comprised Benin and the Democratic Republic of the Congo; Nigeria and Gabon; Eritrea, Sudan and Libya; as well as Benin, Togo, Ethiopia, the Philippines and Iran

^b^Various destinations comprised Mali and the Ivory Coast

The time between returning from the travel destinations and the onset of clinical symptoms leading to the diagnosis of malaria (latency period) was documented for 52 patients (27 with *P. o. curtisi*, 25 with *P. o. wallikeri*). Latency periods were 150.9 ± 232.1 days (mean ± SD) with a median of 56.0 days (IQR = 153.5 days) for patients with *P. o. curtisi* and 105.7 ± 127.9 days (mean ± SD) with a median of 58 days (IQR = 98.0 days) for patients with *P. o. wallikeri* (P = 0.80) (Table 2, Additional file 1).

Thrombocyte count was available for 10 patients with *P. o. curtisi* and 9 patients with *P. o. wallikeri*, with values of 135.1 ± 56.8/µL (mean ± SD) with a median of 138.5/µL (IQR = 105.8/µL) and 183.1 ± 114.2/µL (mean ± SD) with a median of 152.0/µL (IQR = 155.0/µL), respectively (P = 0.40) (Table 2, Additional file 1).

## Discussion

The study compared two recently published real-time PCR protocols for the identification and differentiation of *P. o. curtisi* and *P. o. wallikeri* in clinical samples. Results of parasite subtyping were correlated with patient characteristics. All patients were travellers returning to Germany from countries known to be endemic for malaria. The results further confirmed previous findings about the high specificity of PCR for the diagnosis of non-falciparum malaria at species level, as several misidentifications during the initial microscopic assessment were corrected. In addition, both real-time PCR protocols that were compared in this study proved to be suitable for the identification of *P. o. curtisi* and *P. o. wallikeri* with 100% concordance. The protocol of Bauffe et al. [18] was slightly more sensitive than that of Calderaro et al. [32, 33], as suggested by lower Ct values and by a case of failed detection of *P. o. wallikeri* in a post-treatment control sample. Another advantage of the Bauffe protocol was that the assay can be run as a one-well-duplex PCR, while the Calderaro protocol requires two separate PCR assays to distinguish *P. o. curtisi* and *P. o. wallikeri*. Accordingly, the relatively more sensitive and convenient duplex PCR by Bauffe et al. [18] is the preferred test in this patient population.

Of note, *P. ovale* spp. infections frequently occur in asymptomatic individuals in high endemicity settings [40]. Underlying acquired resistance phenomena have been described as early as in the late 1930s [41, 42]. In the here-described study, only patients were included whose clinical symptoms had triggered diagnostic testing for malaria. Accordingly, no conclusions can be drawn on the diagnostic performance of the applied PCR approaches in patients with completely asymptomatic *P. ovale* spp. infections. From previous studies, however, generally superior sensitivity of PCR compared to microscopy [43–45] and alternative modern approaches like mass spectrometry [46] for malaria screening are well documented. Especially, species-specific malaria PCR is considered as more reliable in detecting mixed plasmodial infections in particular in cases with significant differences in density of the different parasite species [44, 47, 48]. Further, PCR is a reliable tool if suboptimal pre-analytic conditions do not allow parasite microscopy, e.g. in case of hemolytic blood [49].

With regard to *P. ovale* spp. epidemiology, the here-described assessment clearly confirmed the co-occurrence of *P. o. curtisi* and *P. o. wallikeri* in a wide range of malaria-endemic countries in Sub-Saharan Africa, as already reported [3, 4]. In contrast to previous reports [13–15], the findings shown here did not support the suggestion of earlier onsets of clinical disease due to *P. o. wallikeri* compared with *P. o. curtisi* [13–15]. Of note, latency could only be assessed for 52 out of 77 patients (67.5%). Likewise, significant differences in thrombocyte counts were not confirmed. Differences in thrombocyte counts have been reported earlier and were used as an indicator for differences in disease severity. Admittedly, thrombocyte count was available for 19 patients (24.7%) only. Altogether, the here-described study comprised only half as many samples as the assessment by Nolder et al. [15] but it was of similar size to the multi-national study of Rojo-Marcos et al. [14] comprising 79 patients. However, the latter multicentre study [14] was prospective and thus more robust, while the interpretation of the here-described study is limited by its retrospective design. As further limitations, patient characteristics and travel information were documented only for subsets of patients and information on the proportions of travellers visiting friend and relatives as well as of immigrants were no available. Next to this, details on ethnicity, prophylaxis and therapy could not be provided.

## Conclusions

In conclusion, the study confirmed the suitability of two real-time PCR protocols for the discrimination of *P. ovale* spp. into *P. o. curtisi* and *P. o. wallikeri* as well as the sympatric occurrence of the two species in a wide range of countries in sub-Saharan Africa. In contrast, significant differences in latency periods or thrombocyte counts between patients infected with either of the two parasite species as described previously, were not supported. Admittedly, interpretation of those findings is limited by the low number of available datasets. The *P. ovale* spp. malaria cases reported here were associated with low parasitaemia. Many of them were only PCR-positive, so they probably had submicroscopic malaria. Taken together, from a medical point of view, the results presented here do not support the necessity of *P. ovale* species differentiation into *P. ovale curtisi* and *P. ovale wallikeri* for the clinical management of *P. ovale* spp. malaria patients. Next to this, the data shown here stress the importance of PCR for the diagnosis of *P. ovale* spp. malaria.

## Additional files

**Additional file 1.** Details of patients and samples.

**Additional file 2.** Oligonucleotides used in the *P. ovale* spp. differentiation PCR platforms.

**Additional file 3.** Graphical distribution of *P. ovale curtisi* (red dots) and *P. ovale wallikeri* (yellow dots) cases according to countries in which infections were most likely acquired.

## Data Availability

All data generated or analysed during this study are included in this published article and its additional files.

## Abbreviations

°C: degree centigrade
Ct: cycle threshold
BNITM: Bernhard Nocht Institute for Tropical Medicine
EDTA: ethylenediaminetetraacetic acid
Inc.: incorporated
INR: international normalized ratio
IQR: interquartile range
µL: microlitre
PCR: polymerase chain reaction
pmol: picomole
qPCR: real-time PCR
rRNA: ribosomal ribonucleic acids
SD: standard deviation
spp.: species (plural)
