# Supplementary material for: A comparison of two PCR protocols for the differentiation of Plasmodium ovale species and implications for clinical management in travellers returning to Germany: a 10-year cross-sectional study
Source: Malar J. 2019 Aug 9;18:272. doi: 10.1186/s12936-019-2901-0 (PMC6688346; doi:10.1186/s12936-019-2901-0)
Supplement: Supplementary file 1 — Additional file 1. Details of patients and samples. [file 12936_2019_2901_MOESM1_ESM.docx]

**Additional material 1:** Details of patients and samples.

| Sample number* | Gender | Age (years) | Region of likely malaria acquisition | Time between return to Germany and diagnosis of malaria (days) | Microscopic result (genus/species) | Microscopic parasitemia (parasites/µL or % of infected red blood cells) | Result of multiplex-PCR targeting *P. falciparum*, *P. malariae*, *P. ovale* spp., *P. vivax*, and *P. knowlesi* | Ct-value of PCR for *P. ovale curtisi* according to Bauffe et al. | Ct-value of PCR for *P. ovale wallikeri* according to Bauffe et al. | Ct-value of PCR for *P. ovale curtisi* according to Calderaro et al. | Ct-value of PCR *for P. ovale wallikeri* according to Calderaro et al. | Thrombo-cyte count (cells/µL) |
| --- | --- | --- | --- | --- | --- | --- | --- | --- | --- | --- | --- | --- |
| 1 | M | 39 | Benin | 270 | *P. vivax* | 4,960 / µL | *P. ovale* spp. | negative | **22** | negative | **27** | 238 |
| 1.1* | M | 39 | Benin | 270 | PCR only | PCR only | *P. ovale* spp. | negative | **22** | negative | **25** | 238 |
| 2 | M | NA | Uganda | 156 | PCR only | PCR only | *P. ovale* spp. | **24** | negative | **26** | negative | NA |
| 3 | M | 16 | NA | NA | *P. ovale* spp. | 5,520 / µL | *P. ovale* spp. | **23** | negative | **26** | negative | NA |
| 4 | M | 22 | Equatorial Guinea | 172 | PCR only | PCR only | *P. ovale* spp. | **23** | negative | **25** | negative | NA |
| 5 | F | 23 | Malawi | NA | *Plasmodium* spp. | 680 / µL | *P. ovale* spp. | **26** | negative | **29** | negative | NA |
| 6 | M | 51 | Benin, Democratic Republic of the Congo | 44 | *Plasmodium vivax/ovale* spp. | 1,584 / µL | *P. ovale* spp. | **24** | negative | **26** | negative | 197 |
| 7 | M | 40 | Ivory Coast | 375 | *P. ovale* spp. | 1,080 / µL | *P. ovale* spp. | negative | **23** | negative | **25** | 131 |
| 8 | M | 15 | NA | NA | *Plasmodium* spp. | 600 / µL | *P. ovale* spp. | **23** | negative | **24** | negative | NA |
| 9 | F | 15 | Sierra Leone | 65 | *P. ovale* spp. | 213 / µL | *P. ovale* spp. | negative | **25** | negative | **28** | NA |
| 10 | F | 35 | Ghana | 0 | PCR only | PCR only | *P. ovale* spp. | negative | **28** | negative | **32** | NA |
| 11 | M | 42 | Uganda | 14 | *Plasmodium vivax/ovale* spp. | 1,360 / µL | *P. ovale* spp. | **24** | negative | **27** | negative | NA |
| 12 | M | 44 | Zambia | 56 | *P. ovale* spp. | 4,720 / µL | *P. ovale* spp. | **23** | negative | **26** | negative | NA |
| 13 | M | 45 | NA | NA | PCR only | PCR only | *P. ovale* spp. | negative | **27** | negative | **31** | NA |
| 14 | M | 45 | Nigeria | 5 | *Plasmodium* spp. | 715 / µL | *P. ovale* spp. | **24** | negative | **26** | negative | 97 |
| 15 | M | 33 | Nigeria, Gabon | 183 | *Plasmodium* spp. | 112 / µL | *P. ovale* spp. | **28** | negative | **31** | negative | NA |
| 16 | M | 55 | Sudan | 10 | PCR only | PCR only | *P. ovale* spp. | negative | **20** | negative | **25** | NA |
| 17 | M | 14 | Cameroon | NA | *P. ovale* spp. | 496 / µL | *P. ovale* spp. | negative | **25** | negative | **29** | NA |
| 18 | F | 22 | Mali, Ivory Coast | 224 | *P. ovale* spp. | 320 / µL | *P. ovale* spp. | negative | **24** | negative | **27** | 261 |
| 19 | F | 34 | Sierra Leone | 309 | *Plasmodium vivax/ovale* spp. | 1,040 / µL | *P. ovale* spp. | **24** | negative | **26** | negative | NA |
| 20 | F | 29 | Nigeria | 115 | *P. ovale* spp. | ≤ 50 / µL^#^ | *P. ovale* spp. | negative | **21** | negative | **25** | NA |
| 21 | M | 51 | Ghana | 2 | *Plasmodium* spp. | niedrige Parasitämie | *P. ovale* spp. | **28** | negative | **30** | negative | NA |
| 22 | M | 18 | Ivory Coast | 45 | *P. ovale* spp. | 928 / µL | *P. ovale* spp. | **23** | negative | **26** | negative | 203 |
| 23 | M | 21 | NA | NA | *P. vivax* | 3,200 / µL | *P. ovale* spp. | **23** | negative | **26** | negative | NA |
| 23.1* | M | 21 | NA | NA | PCR only | PCR only | *P. ovale* spp. | **22** | negative | **24** | negative | NA |
| 24 | M | 6 | Cameroon | 58 | *P. ovale* spp. | - | *P. ovale* spp. | negative | **23** | negative | **27** | NA |
| 25 | F | 22 | Ghana | NA | *P. ovale* spp. | 0.1% | *P. ovale* spp. | negative | **26** | negative | **28** | NA |
| 26 | M | 36 | Liberia | NA | PCR only | PCR only | *P. ovale* spp. | **28** | negative | **30** | negative | NA |
| 27 | M | 20 | Uganda | 214 | *Plasmodium vivax/ovale* spp. | 1,200 / µL | *P. ovale* spp. | negative | **23** | negative | **31** | NA |
| 28 | M | 25 | Eritrea, Sudan, Libya | 473 | PCR only | PCR only | *P. ovale* spp. | **28** | negative | **29** | negative | 119 |
| 29 | M | 18 | Kenia | 7 | PCR only | PCR only | *P. ovale* spp. | **25** | negative | **28** | negative | NA |
| 30 | M | 24 | NA | NA | *Plasmodium vivax/ovale* spp. | 18,960 / µL | *P. ovale* spp. | **19** | negative | **21** | negative | NA |
| 31 | F | 21 | Tanzania | 85 | *Plasmodium* spp. | 187 / µL | *P. ovale* spp. | negative | **25** | negative | **27** | 234 |
| 32 | NA | 26 | NA | NA | *Plasmodium* spp. | 1,280 / µL | *P. ovale* spp. | **23** | negative | **26** | negative | NA |
| 33 | M | 46 | Nigeria | 91 | *Plasmodium* spp. | 288 / µL | *P. ovale* spp. | **25** | negative | **25** | negative | NA |
| 34 | M | 52 | Ghana | 94 | *P. vivax* | 3,000 / µL | *P. ovale* spp. | **23** | negative | **23** | negative | 163 |
| 35 | M | 68 | Ghana | 32 | *Plasmodium* spp. | 416 / µL | *P. ovale* spp. | **26** | negative | **27** | negative | NA |
| 36 | F | 40 | Cameroon | 24 | *Plasmodium* spp. | 128 / µL | *P. ovale* spp. | negative | **28** | negative | **31** | 81 |
| 37 | F | 19 | Cameroon | NA | *Plasmodium* spp. | 720 / µL | *P. ovale* spp. | negative | **25** | negative | **29** | NA |
| 38 | F | 63 | Ghana, Liberia | 30 | *Plasmodium* spp. | 69 / µL | *P. ovale* spp. | **28** | negative | **28** | negative | 78 |
| 39 | M | 18 | NA | NA | *Plasmodium* spp. | ≤ 50 / µL^#^ | *P. ovale* spp. | **24** | negative | **26** | negative | NA |
| 40 | F | 46 | Cameroon | 50 | PCR only | PCR only | *P. ovale* spp. | negative | **37** | negative | **45** | NA |
| 40.1* | F | 46 | Cameroon | 50 | PCR only | PCR only | *P. ovale* spp. | negative | **37** | negative | negative | NA |
| 41 | M | 17 | NA | NA | PCR only | PCR only | *P. ovale* spp. | negative | **26** | negative | **29** | NA |
| 42 | M | 39 | Nigeria | 6 | *Plasmodium* spp. | 480 / µL | *P. ovale* spp. | **26** | negative | **27** | negative | 61 |
| 43 | M | 48 | Cameroon | 19 | PCR only | PCR only | *P. ovale* spp. | negative | **22** | negative | **24** | 56 |
| 44 | F | 41 | Gabon | 3 | *Plasmodium* spp. | ≤ 50 / µL^#^ | *P. ovale* spp. | negative | **26** | negative | **29** | NA |
| 45 | F | 53 | Cameroon | 124 | PCR only | PCR only | *P. ovale* spp. | **23** | negative | **24** | negative | NA |
| 46 | M | 53 | Ghana | NA | PCR only | PCR only | *P. ovale* spp. | negative | **24** | negative | **27** | NA |
| 47 | M | 53 | Uganda | 6 | *Plasmodium vivax/ovale* spp. | 4,040 / µL | *P. ovale* spp. | **23** | negative | **23** | negative | 204 |
| 48 | F | 5 | Ghana | NA | PCR only | PCR only | *P. ovale* spp. | negative | **26** | negative | **29** | NA |
| 49 | F | 19 | Somalia | 125 | *P. ovale* spp. | 1% | *P. ovale* spp. | **22** | negative | **23** | negative | NA |
| 50 | F | 59 | NA | NA | PCR only | PCR only | *P. ovale* spp. | negative | **25** | negative | **21** | NA |
| 51 | F | 53 | Papua New Guinea | 115 | PCR only | PCR only | *P. ovale* spp. | negative | **23** | negative | **28** | NA |
| 52 | M | 17 | Tanzania | 7 | *Plasmodium vivax/ovale* spp. | 13,000 / µL | *P. ovale* spp. | **22** | negative | **24** | negative | NA |
| 53 | F | 26 | Cameroon | 959 | PCR only | PCR only | *P. ovale* spp. | **23** | negative | **25** | negative | NA |
| 54 | M | 21 | NA | NA | PCR only | PCR only | *P. ovale* spp. | negative | **22** | negative | **24** | NA |
| 55 | M | 52 | Togo | 1 | *Plasmodium* spp. | 320 / µL | *P. ovale* spp. | negative | **27** | negative | **29** | 412 |
| 56 | M | 31 | Kenia | 31 | *Plasmodium vivax/ovale* spp. | 19,920 / µL | *P. ovale* spp. | negative | **19** | negative | **24** | 152 |
| 57 | M | 8 | NA | NA | *Plasmodium vivax/ovale* spp. | 2,360 / µL | *P. ovale* spp. | **23** | negative | **24** | negative | NA |
| 58 | M | 30 | Tanzania | 31 | PCR only | PCR only | *P. ovale* spp. | negative | **26** | negative | **30** | NA |
| 59 | M | 38 | Cameroon | 2 | PCR only | PCR only | *P. ovale* spp. | **26** | negative | **27** | negative | 71 |
| 60 | M | 22 | Ghana | 7 | PCR only | PCR only | *P. ovale* spp. | negative | **37** | negative | **39** | NA |
| 61 | M | 21 | Malawi | NA | PCR only | PCR only | *P. ovale* spp. | negative | **22** | negative | **25** | NA |
| 62 | F | 26 | NA | NA | PCR only | PCR only | *P. ovale* spp. | **22** | negative | **24** | negative | NA |
| 63 | M | 34 | Burkina Faso | 454 | *Plasmodium* spp. | 520 / µL | *P. ovale* spp. | negative | **26** | negative | **30** | NA |
| 64 | M | 30 | Guinea | 287 | PCR only | PCR only | *P. ovale* spp. | **26** | negative | **28** | negative | 158 |
| 64.1* | M | 30 | Guinea | 287 | *Plasmodium vivax/ovale* spp. | 1,280 / µL | *P. ovale* spp. | **29** | negative | **30** | negative | 158 |
| 65 | M | 8 | Guinea | 20 | PCR only | PCR only | *P. ovale* spp. | **31** | negative | **33** | negative | NA |
| 66 | M | 36 | Liberia | 735 | PCR only | PCR only | *P. ovale* spp. | **30** | negative | **33** | negative | NA |
| 67 | F | 18 | NA | NA | *Plasmodium vivax/ovale* spp. | 17,400 / µL | *P. ovale* spp. | **21** | negative | **24** | negative | NA |
| 68 | M | 18 | Ghana | NA | PCR only | PCR only | *P. ovale* spp. | **35** | negative | **37** | negative | NA |
| 69 | M | 20 | Nigeria | 62 | PCR only | PCR only | *P. ovale* spp. | negative | **22** | negative | **25** | NA |
| 70 | M | 48 | NA | NA | PCR only | PCR only | *P. ovale* spp. | negative | **25** | negative | **27** | NA |
| 71 | F | 23 | Ivory Coast | 314 | PCR only | PCR only | *P. ovale* spp. | negative | **17** | negative | **28** | NA |
| 72 | M | NA | Nigeria | NA | PCR only | PCR only | *P. ovale* spp. | **29** | negative | **32** | negative | NA |
| 73 | M | 30 | Uganda | 17 | *P. ovale* spp. | 2,960 / µL | *P. ovale* spp. | negative | **20** | negative | **24** | NA |
| 74 | M | 20 | Ghana | 83 | PCR only | PCR only | *P. ovale* spp. | negative | **35** | negative | **36** | 83 |
| 75 | F | 47 | NA | NA | *Plasmodium vivax/ovale* spp. | 5,420 / µL | *P. ovale* spp. | **22** | negative | **25** | negative | NA |
| 76 | M | 20 | Cameroon | 16 | PCR only | PCR only | *P. ovale* spp. | negative | **25** | negative | **28** | NA |
| 76.1* | M | 20J | Cameroon | 16 | PCR only | PCR only | *P. ovale* spp. | negative | **25** | negative | **29** | NA |
| 77 | M | NA | Iran, Benin, Togo, Ethiopia, the Philippines | 91 | *Plasmodium vivax/ovale* spp. | 1% | *P. ovale* spp. | **24** | negative | **25** | negative | NA |

*n.1 describes a second sample from the same patients.

NA = not available. PCR only = Microscopic assessment had not been requested in the diagnostic workflow. M = male, F = female.
