# Supplementary material for: A comparison of two PCR protocols for the differentiation of Plasmodium ovale species and implications for clinical management in travellers returning to Germany: a 10-year cross-sectional study
Source: Malar J. 2019 Aug 9;18:272. doi: 10.1186/s12936-019-2901-0 (PMC6688346; doi:10.1186/s12936-019-2901-0)
Supplement: Supplementary file 2 — Additional file 2. Oligonucleotides used in the P. ovale spp. differentiation PCR platforms. [file 12936_2019_2901_MOESM2_ESM.docx]

Additional material 2: Oligonucleotides used in the *P. ovale* spp. differentiation PCR platforms.

| Platform 1: Oligonucleotide sequences used for the duplex real-time PCR for the discrimination of *P. ovale curtisi* and *P. ovale wallikeri* according to Bauffe et al. (18) | | |
| --- | --- | --- |
| Common forward primer POF cur/wal - F | | 5′-ATA-AAC-TAT-GCC-GAC-TAG-GTT-3′ |
| Common reverse primer POF cur/wal – R | | 5′-ACT-TTG-ATT-TCT-CAT-AAG-GTA-CT-3′ |
| Probe POF curti – T targeting *P. ovale curtisi* | | 5′-TTC-CTT-TCG-GGG-AAA-TTT-CTT-AGA-3′ |
| POF walli – T targeting *P. ovale wallikeri* | | 5′-AAT-TCC-TTT-TGG-AAA-TTT-CTT-AGA-TTG-3′ |
| *P. ovale wallikeri* positive control plasmid insert (GenBank: MG241227.1) | | 5′-AAT-CTT-AAC-CAT-AAA-CTA-TGC-CGA-CTA-GGT-TTT-GGA-TGA-AAG-ATT-TTT-AAA-TAA-GAA-AAT-TCC-TTT-TGG-AAA-TTT-CTT-AGA-TTG-CTT-CCT-TCA-GTA-CCT-TAT-GAG-AAA-TCA-AAG-TCT-TTG-GGT-TC-3′ |
| *P. ovale curtisi* positive control plasmid insert (GenBank: KX672023.1) | | 5′-AAT-CTT-AAC-CAT-AAA-CTA-TGC-CGA-CTA-GGT-TTT-GGA-TGA-AAC-ATT-TTT-AAA-TAA-GAA-AAT-TCC-TTT-CGG-GGA-AAT-TTC-TTA-GAT-TGC-TTC-TTT-CAG-TAC-CTT-ATG-AGA-AAT-CAA-AGT-CTT-TGG-GTT-C-3′ |
| Platform 2: Oligonucleotide sequences used for the two simplex real-time PCRs for *P. ovale curtisi* and *P. ovale wallikeri* according to Calderaro et al. (31, 32) | | |
| 2.1: Real-time PCR for *P. ovale curtisi* | | |
| Forward primer OVA curti - F | | 5′-TTT-TGA-AGA-ATA-CAT-TAG-GAT-ACA-ATT-AAT-G-3′ |
| Reverse primer OVA cur/wal - R | | 5′-CAT-CGT-TCC-TCT-AAG-AAG-CTT-TAC-AAT-3′ |
| Probe OVA curti -T | | 5′-CCT-TTT-CCC-TAT-TCT-ACT-TAA-TTC-GCA-ATT-CAT-G-3′ |
| *P. ovale curtisi* positive control plasmid insert (GenBank: AB182489.1) | | 5′-AAT-TTG-CTT-ATT-TTG-AAG-AAT-ACA-TTA-GGA-TAC-AAT-TAA-TGT-GTC-CTT-TTC-CCT-ATT-CTA-CTT-AAT-TCG-CAA-TTC-ATG-CTG-TTT-CTC-TTT-TGC-ATA-GGA-ATG-TAT-TCG-TTT-GAT-TGT-AAA-GCT-TCT-TAG-AGG-AAC-GAT-GTG-TGT-CTA-AC-3′ |
| 2.2: Real-time PCR for *P. ovale wallikeri* | | |
| OVA walli - F | 5′-TTT-TGA-AGA-ATA-TAT-TAG-GAT-ACA-TTA-TAG-3′ | |
| OVA cur/wal - R | 5′-CAT-CGT-TCC-TCT-AAG-AAG-CTT-TAC-AAT-3′ | |
| OVA walli - T | 5′-CCT-TTT-CCC-TTT-TCT-ACT-TAA-TTC-GCT-ATT-CAT-G-3′ | |
| *P. ovale wallikeri* positive control plasmid insert (GenBank: AJ001527.1) | 5′-AAT-TTG-CTT-ATT-TTG-AAG-AAT-ATA-TTA-GGA-TAC-ATT-ATA-GTG-TCC-TTT-TCC-CTT-TTC-TAC-TTA-ATT-CGC-TAT-TCA-TGC-TGT-TTC-TTT-TTT-GTG-TAG-GAA-TGT-ATT-CGT-TTG-ATT-GTA-AAG-CTT-CTT-AGA-GGA-ACG-ATG-TGT-GTC-TAA-C-3′ | |
