# Supplementary material for: A comparison of two PCR protocols for the differentiation of Plasmodium ovale species and implications for clinical management in travellers returning to Germany: a 10-year cross-sectional study
Source: Malar J. 2019 Aug 9;18:272. doi: 10.1186/s12936-019-2901-0 (PMC6688346; doi:10.1186/s12936-019-2901-0)

Additional material 3: Graphical distribution of *P. ovale curtisi* (red dots) and *P. ovale wallikeri* (yellow dots) cases according to countries in which infections were most likely acquired.


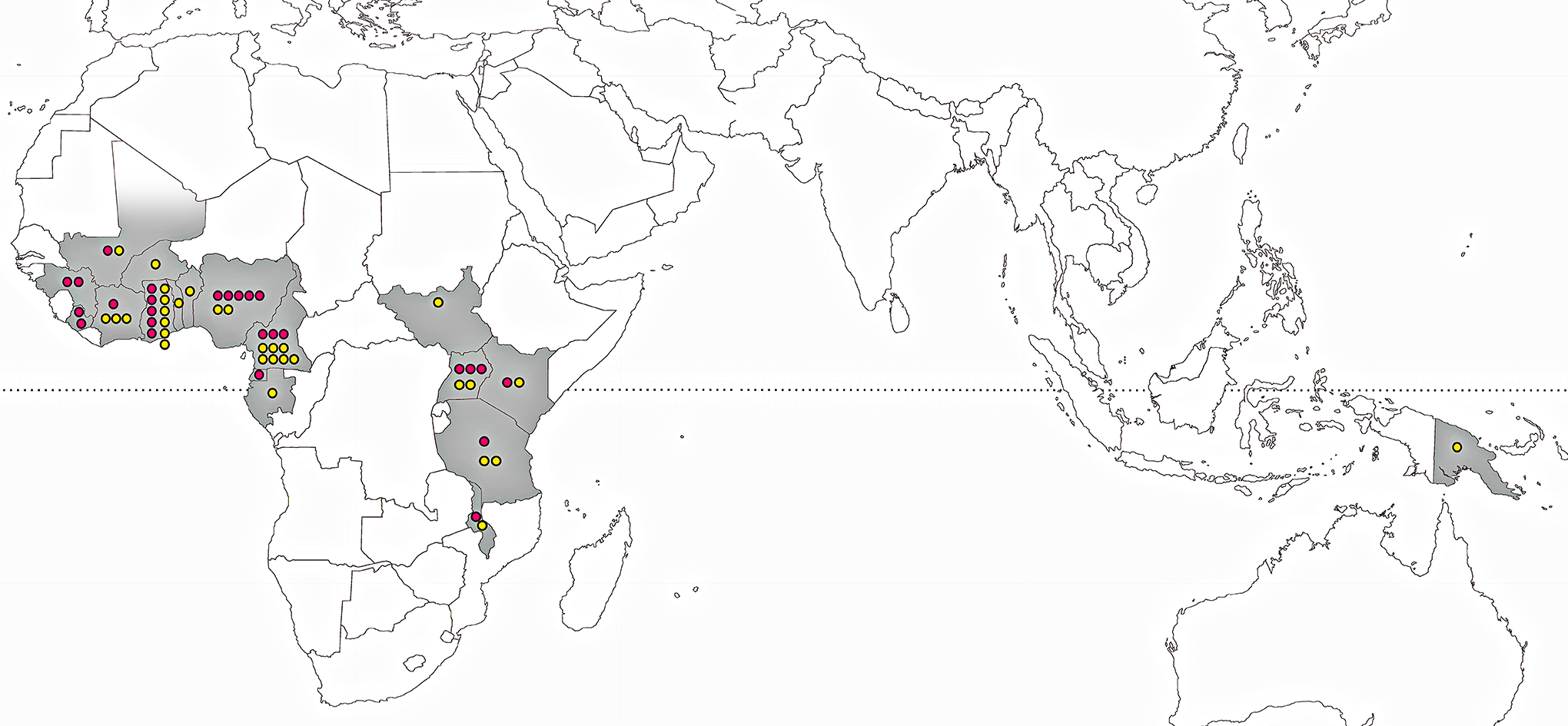

Supplement: Supplementary file 3 — Additional file 3. Graphical distribution of P. ovale curtisi (red dots) and P. ovale wallikeri (yellow dots) cases according to countries in which infections were most likely acquired. [file 12936_2019_2901_MOESM3_ESM.docx]
